# Synergistic Activity of the Human Lactoferricin-Derived Peptide hLF1-11 in Combination with Caspofungin against *Candida* Species

**DOI:** 10.1128/spectrum.01240-22

**Published:** 2022-07-25

**Authors:** Roberta Fais, Cosmeri Rizzato, Iacopo Franconi, Arianna Tavanti, Antonella Lupetti

**Affiliations:** a Department of Translational Research and of New Technologies in Medicine and Surgery, University of Pisa, Pisa, Italy; b Department of Biology, University of Pisa, Pisa, Italy; University of Iowa Hospitals and Clinics

**Keywords:** hLF1-11, caspofungin, synergism, *Candida* spp., biofilm

## Abstract

*Candida* species are the main fungal opportunistic pathogens causing systemic infections that are often associated with drug resistance and biofilm production on medical devices. The pressing need for new antifungal agents led to an increased interest in the use of combination therapies. The present study was aimed at investigating potential synergistic activity of the human lactoferrin-derived hLF1-11 peptide with caspofungin against caspofungin-resistant or -susceptible C. albicans, C. parapsilosis, and C. glabrata strains. Synergism was evaluated by the checkerboard assay, measuring cellular metabolic activity against *Candida* planktonic and sessile cells. A fractional inhibitory concentration (FIC) index of ≤0.5 was interpreted as synergy. Synergism was evaluated by killing assays on planktonic cells. A cell viability assay was performed with biofilm formation inhibition and preformed biofilm. Synergy for killing and viability assays was defined as a ≥2-log-CFU/mL reduction in comparison with the most active constituent. hLF1-11 and caspofungin exerted (i) synergistic effects against planktonic cells of all the tested strains, yielding drastic caspofungin MIC reduction, (ii) synergistic effects on the inhibition of biofilm formation against biofilm producer strains, yielding caspofungin BIC reduction, and (iii) synergistic effects on preformed biofilm assessed by measuring metabolic activity (FIC range, 0.28 to 0.37) against biofilm-producing strains and by cell viability assay in C. albicans SC5314. The synergistic effect observed between caspofungin and hLF1-11 against *Candida* spp. is of potential clinical relevance, representing a promising novel approach to target caspofungin-resistant *Candida* species infections. Further studies elucidating the mechanisms of action of such a synergistic effect are needed.

**IMPORTANCE** The present study describes a synergistic effect between a conventional antifungal drug, caspofungin, and a synthetic peptide derived from human lactoferrin, hLF1-11, against *Candida* species. These yeasts are able to cause severe systemic fungal infections in immunocompromised hosts. In addition, they can form biofilms in medical implanted devices. Recently, caspofungin-resistant *Candida* strains have emerged, thus highlighting the need to develop different therapeutic strategies. In *in vitro* studies, this drug combination is able to restore sensitivity to caspofungin in caspofungin-resistant strains of *Candida* species, both in free-living cells and in cells organized in biofilms. This synergism could represent a promising novel approach to target infections caused by caspofungin-resistant *Candida* species.

## INTRODUCTION

*Candida* species are the main fungal opportunistic pathogens in current clinical practice. Over 250.000 immunocompromised patients/year are affected by invasive candidiasis worldwide, causing 50.000 deaths (1). Candidemia represents the fourth most common nosocomial bloodstream infection (1–3). C. albicans, C. glabrata, C. parapsilosis, C. tropicalis, and C. krusei are responsible for >90% of candidemia, the most common being C. albicans (<50% of all candidemia in the United States), followed by C. glabrata (33%) and C. parapsilosis (15%) (4–7). In Europe, the prevalence of C. albicans from blood culture differs among countries, ranging from 43.63% in Italy to 68% in Norway (4, 5, 8). With regard to the hosts’ clinical characteristics, C. glabrata seems to affect older patients and subjects with malignancies, while C. parapsilosis is usually catheter-related, affecting newborns and intensive care unit (ICU) patients (9, 10).

Despite the availability of new drugs, the mortality rate for candidemia is still high, with no significant improvement on prognosis (11, 12). This may be due to the aforementioned poor clinical conditions of patients as well as to the development of drug-resistant *Candida* isolates. Clinical resistance reflects the inability to eradicate infections caused by microorganisms that have shown *in vitro* susceptibility to current antifungal therapy (12). To this point, biofilm formation plays a pivotal role in the development of clinical resistance (13). Biofilms are a community of multiple microbes surrounded by extracellular polysaccharide substance growing into a three-dimensional structure that enables them to adhere to host tissues and survive exposure to antifungal agents (13).

Due to the development of fluconazole-resistant *Candida* spp. and further increases in coresistance to echinocandin, as described in 5.5% to 7.6% of fluconazole-resistant C. glabrata isolates (6), new antifungal agents/therapeutic strategies are an unmet need in the treatment of invasive fungal infections (i.e., antimicrobial proteins/peptides) (14–16). Lactoferrin is a 77-kDa iron-binding glycoprotein found in the exocrine secretion of mammals, blood, and neutrophil-specific granules. After acid pepsinolysis, lactoferrin releases lactoferricin H, which is composed by two cationic domains (residues 2 to 5 and 28 to 31) in its N terminus. A synthetic peptide containing the first 11 residues of lactoferricin, further referred to as hLF1-11, shows direct antimicrobial and antibiofilm activities and synergistic effects with conventional antimicrobials (10, 14, 15, 17–19). The present study was aimed at investigating a potential synergistic activity between hLF1-11 and caspofungin against reference strains and clinical isolates of C. albicans, C. parapsilosis, and C. glabrata, selected on the basis of caspofungin sensitivity.

(This study was presented in part at the 47th Congress of the Italian Society of Microbiology, 2019 [poster no. 114], and 49th Congress of the Italian Society of Microbiology [virtual], 2021.)

## RESULTS

### Synergistic activity of hLF1-11 and caspofungin determined with *Candida* planktonic cells.

The synergistic activity of hLF1-11 and caspofungin was evaluated by the checkerboard method. The MIC values of caspofungin and hLF1-11 against susceptible and resistant *Candida* strains are reported in Table 1. hLF1-11 MIC values for C. albicans and C. glabrata ranged from 22 to 88 μg/mL, and that for C. parapsilosis was 22 μg/mL. Caspofungin MIC values for C. albicans ranged from 0.25 to 2 μg/mL, and those for C. glabrata and C. parapsilosis ranged from 1 to 2 μg/mL.

**TABLE 1:** Checkerboard assay on planktonic cells

Strain	MIC (μg/mL)			Mean lowest FIC
	Caspofungin	hLF1-11	Caspofungin–hLF1-11	
CA-CR	1–2	44–88	0.06/11	0.29
CG-C1	1	44	0.0015/2.75	0.19
CG-C2	1–2	88	0.12/22	0.22
CP7	2	22	0.25/2.75	0.24
SC5314	0.25–0.5	22–44	0.0075/1.37	0.13
ATCC 22019	1–2	22	0.25/2.75	0.33

The checkerboard results revealed synergistic effects between hLF1-11 and caspofungin on planktonic cells of all the tested clinical isolates and reference strains, with the mean lowest fractional inhibitory concentration (FIC) values reported in Table 1. The caspofungin MIC when combined with hLF1-11 was drastically reduced: for caspofungin-resistant C. albicans (CA-CR) from 1 to 2 to 0.06 μg/mL (CA-CR, mean lowest FIC = 0.29), for both caspofungin-resistant C. glabrata strains (CG-C1 and CG-C2) from 1 μg/mL to 0.0015 μg/mL (CG-C1, mean lowest FIC = 0.19) and from 1 to 2 μg/mL to 0.12 μg/mL (CG-C2, mean lowest FIC = 0.22), for C. parapsilosis from 2 μg/mL to 0.25 μg/mL (CP7, mean lowest FIC = 0.24), for the C. albicans reference strain from 0.25 to 0.5 to 0.0075 μg/mL (SC5314, mean lowest FIC = 0.13), and for the C. parapsilosis reference strain from 1 to 2 to 0.25 μg/mL (ATCC 22019, mean lowest FIC = 0.33).

**FIG 1:**
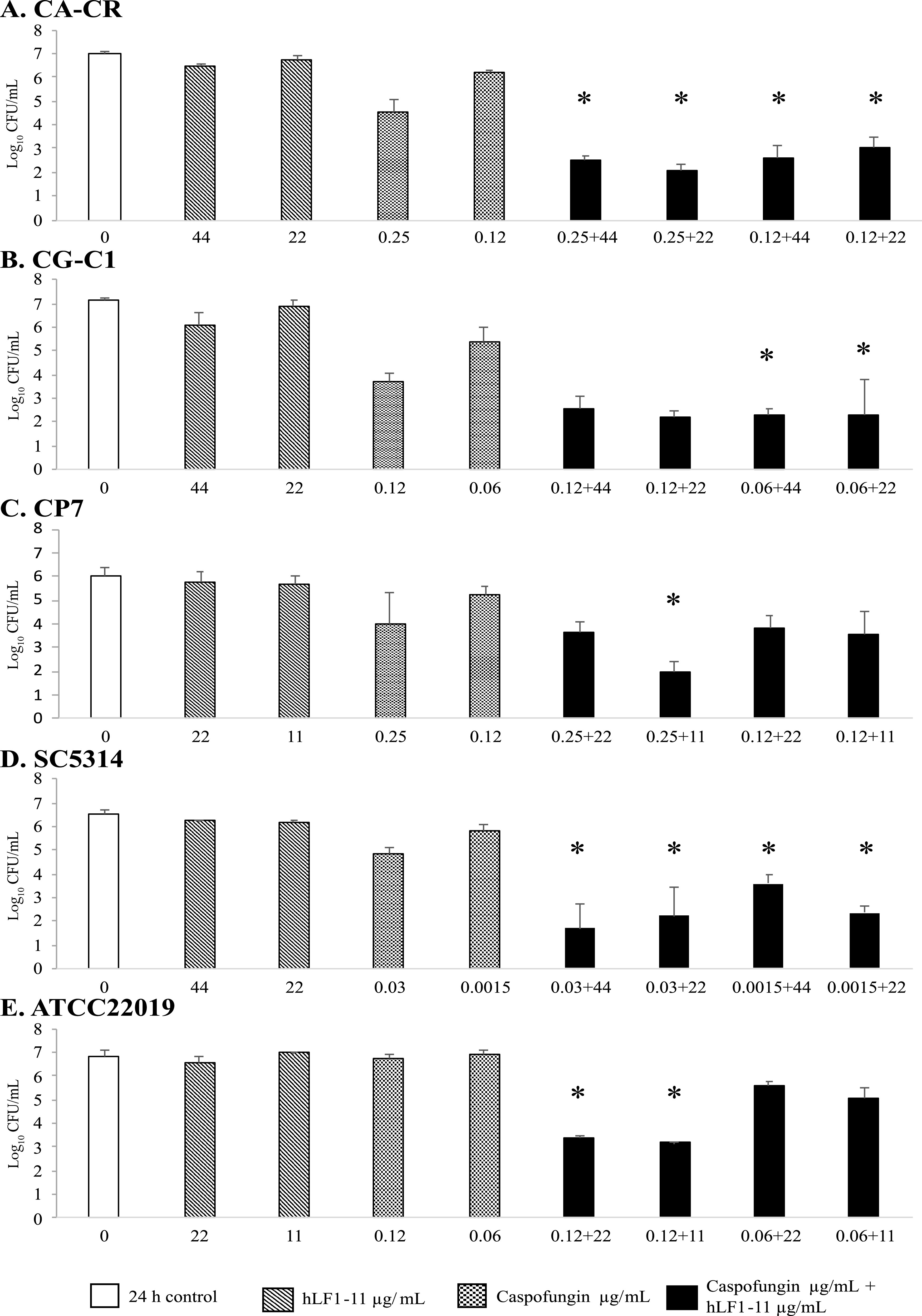
Synergistic activity of hLF1-11 and caspofungin determined on planktonic cells as evaluated by viability assay. Asterisks indicate a >2-log_10_-CFU/mL reduction induced by the combination of hLF1-11 and caspofungin in comparison with its most active constituent. (A) Strain CA-CR; (B) strain CG-C1; (C) strain CP7; (D) strain SC5314; (E) strain ATCC 22019. Open bars, control; diagonally hatched bars, hLF1-11 peptide; dotted bars, caspofungin; solid bars, combinations of caspofungin and hLF1-11.

The killing assay results obtained by hLF1-11 and/or caspofungin on planktonic cells of clinical and reference strains are reported in Figure 1. The concentrations of caspofungin and hLF1-11 tested were chosen among those showing synergisms by the checkerboard method for each strain. For caspofungin-resistant C. albicans (CA-CR), the combination of ineffective concentrations of hLF1-11 and the lowest concentration of caspofungin tested (0.12 μg/mL) induced more than a 3-log reduction in CFU per milliliter in comparison to the most active constituent. In addition, the results revealed a 2-log reduction with 0.25 μg/mL caspofungin in comparison to untreated cells and a synergistic effect when combined with hLF1-11 (Fig. 1A). For caspofungin-resistant C. glabrata (CG-C1), combination treatments with the lowest concentration caspofungin tested showed about 3-log reduction in CFU per milliliter in comparison to the most active constituent (Fig. 1B). For C. parapsilosis (CP7), a synergistic effect was found at 0.25 μg/mL caspofungin and 11 μg/mL hLF1-11 (Fig. 1C). For caspofungin-sensitive C. albicans reference strain SC5314, as expected, synergisms were observed at very low caspofungin concentrations, in the order of nanograms per milliliter (Fig. 1D), whereas for C. parapsilosis ATCC 22019, which is intrinsically less sensitive to caspofungin, the synergistic effect was observed only at 0.12 μg/mL caspofungin (Fig. 1E).

### Synergistic activity of hLF1-11 and caspofungin determined by inhibition of *Candida* biofilm formation.

The checkerboard method was used to evaluate the presence of a synergistic effect between caspofungin and hLF1-11 against *Candida* spp. on biofilm formation inhibition. The results revealed that among the caspofungin-resistant C. glabrata isolates selected, both CG-C1 and CG-C2 were not biofilm-producing strains, and ATCC 22019 was a low biofilm producer (20). Based on these results, checkerboard biofilm inhibition synergy studies were conducted on C. albicans SC5314, caspofungin-resistant C. albicans CA-CR, and C. parapsilosis CP7. The results in terms of biofilm inhibitory concentration (BIC) values for caspofungin and/or hLF1-11 as well as the mean lowest FIC values are reported in Table 2. Synergistic activity was observed for all the tested isolates. When combined with hLF1-11, the caspofungin BIC was reduced for caspofungin-resistant C. albicans (CA-CR) from 1 to 0.03 μg/mL (mean lowest FIC = 0.16), that for C. parapsilosis (CP7) from 1 to 0.12 μg/mL (mean lowest FIC = 0.18), and that for SC5314 from 0.12 to 0.015 μg/mL (mean lowest FIC = 0.15).

**TABLE 2:** Checkerboard inhibition of biofilm formation

Strain	BIC (μg/mL)			Mean lowest FIC
	Caspofungin	hLF1-11	Caspofungin–hLF1-11	
CA-CR	1	88	0.03/11	0.16
CP7	1	22	0.12/1.37	0.18
SC5314	0.12	44	0.015/1.37	0.15

**FIG 2:**
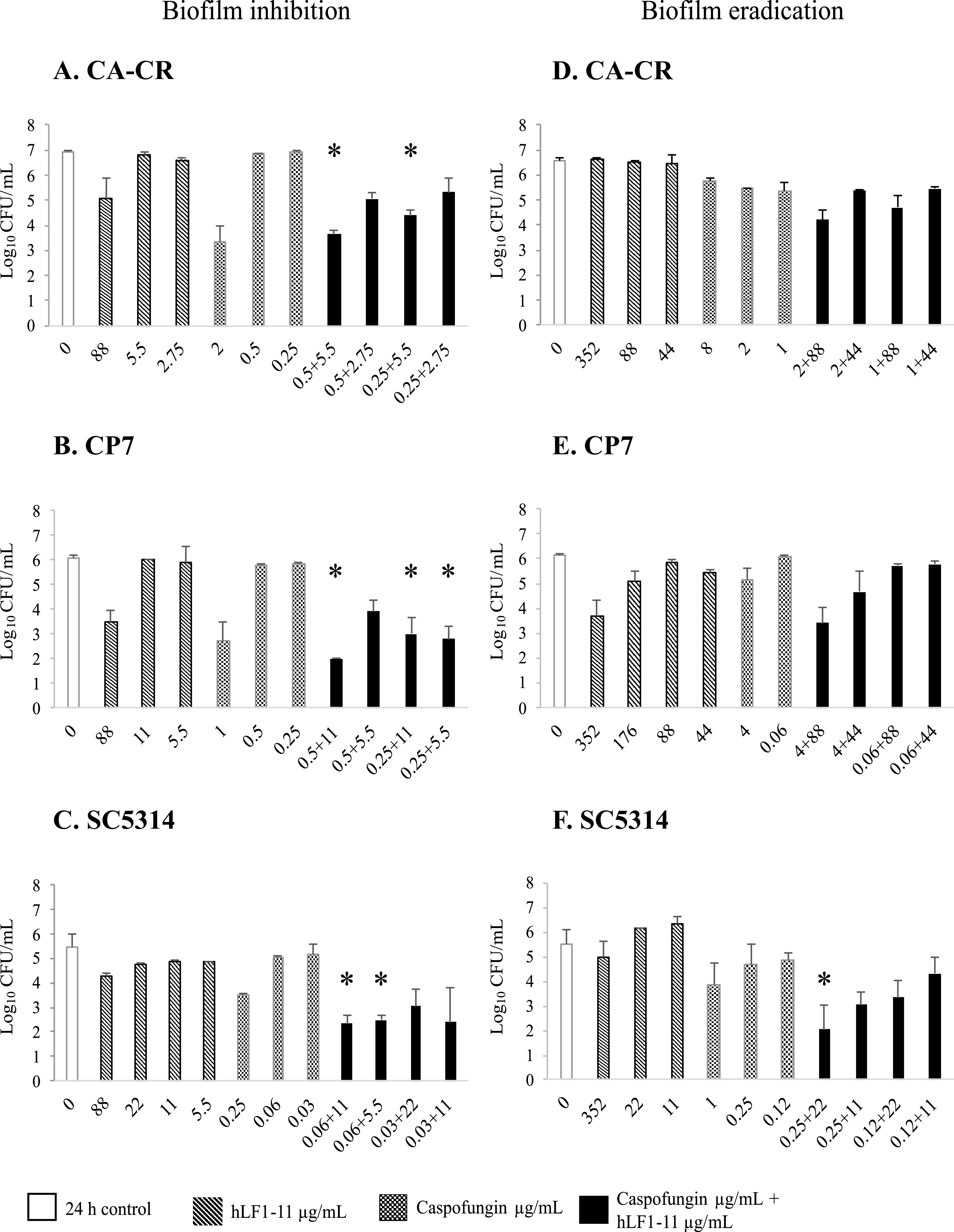
Synergistic activity of hLF1-11 and caspofungin determined by inhibition of *Candida* species biofilm inhibition (A to C) or eradication (D to F) evaluated by viability assay. Asterisks indicate a >2-log_10_-CFU/mL reduction induced by the combination of hLF1-11 and caspofungin in comparison with its most active constituent. (A and D) strain CA-CR; (B and E) strain CP7; (C and F) strain SC5314. Open bars, control; diagonally hatched bars, hLF1-11 peptide; dotted bars, caspofungin; solid bars, combinations of caspofungin and hLF1-11.

*Candida* species sessile cell viability assays were performed on the same strains (Fig. 2A to C). For caspofungin-resistant C. albicans (CA-CR), the combination of ineffective concentrations of hLF1-11 (5.5 μg/mL) and caspofungin (0.5 μg/mL) induced a 3-log reduction in CFU per milliliter in comparison to the most active constituent. In addition, the results revealed a synergistic effect (2-log reduction) with 0.25 μg/mL caspofungin when combined with 5.5 μg/mL hLF1-11 (Fig. 2A). For C. parapsilosis (CP7), more than 3-log reductions in CFU per milliliter were found at 0.5 and 0.25 μg/mL caspofungin plus 11 μg/mL hLF1-11 and at 0.25 μg/mL caspofungin plus 5.5 μg/mL hLF1-11 (Fig. 2B). For caspofungin-sensitive C. albicans reference strain SC5314, as expected, synergisms were observed at concentrations of 0.06 μg/mL caspofungin plus 11 and 5.5 μg/mL hLF1-11 (Fig. 2C).

### Synergistic activity of hLF1-11 and caspofungin determined by *Candida* biofilm eradication.

The checkerboard method was used to evaluate the presence of a synergistic effect between caspofungin and hLF1-11 against *Candida* species on biofilm eradication by measuring its metabolic activity. The BIC values for caspofungin and/or hLF1-11 as well as the mean lowest FIC values are reported in Table 3. Synergistic activity was observed for all the tested isolates. When combined with hLF1-11, the caspofungin BIC was reduced for caspofungin-resistant C. albicans from 0.25 to 0.06 μg/mL, whereas the hLF1-11 BIC was 3-fold reduced (CA-CR, mean lowest FIC = 0.37), for C. parapsilosis, the caspofungin BIC was not reduced, whereas the hLF1-11 BIC was 4-fold reduced (CP7, mean lowest FIC = 0.28), and for the C. albicans reference strain, the caspofungin BIC was reduced from 0.06 to 0.015 μg/mL, whereas the hLF1-11 BIC was 4-fold reduced (SC5314, mean lowest FIC = 0.29).

**TABLE 3:** Checkerboard on mature biofilm

Strain	BIC (μg/mL)			Mean lowest FIC
	Caspofungin	hLF1-11	Caspofungin–hLF1-11	
CA-CR	0.25	64–128	0.06/16	0.37
CP7	2–4	256	2/16	0.28
SC5314	0.06	128–256	0.015/8	0.29

*Candida* species sessile cell viability assays were performed on the same strains (Fig. 2D to F). The results revealed no synergistic activity for both CA-CR (Fig. 2D) and CP7 (Fig. 2E), despite a 4-fold increase in both drug concentrations. For C. albicans reference strain SC5314, a synergistic effect was revealed at 0.25 μg/mL caspofungin plus 22 μg/mL hLF1-11 (Fig. 2F).

### Synergistic activity of hLF1-11 and caspofungin determined by *Candida* biofilm morphology.

**FIG 3:**
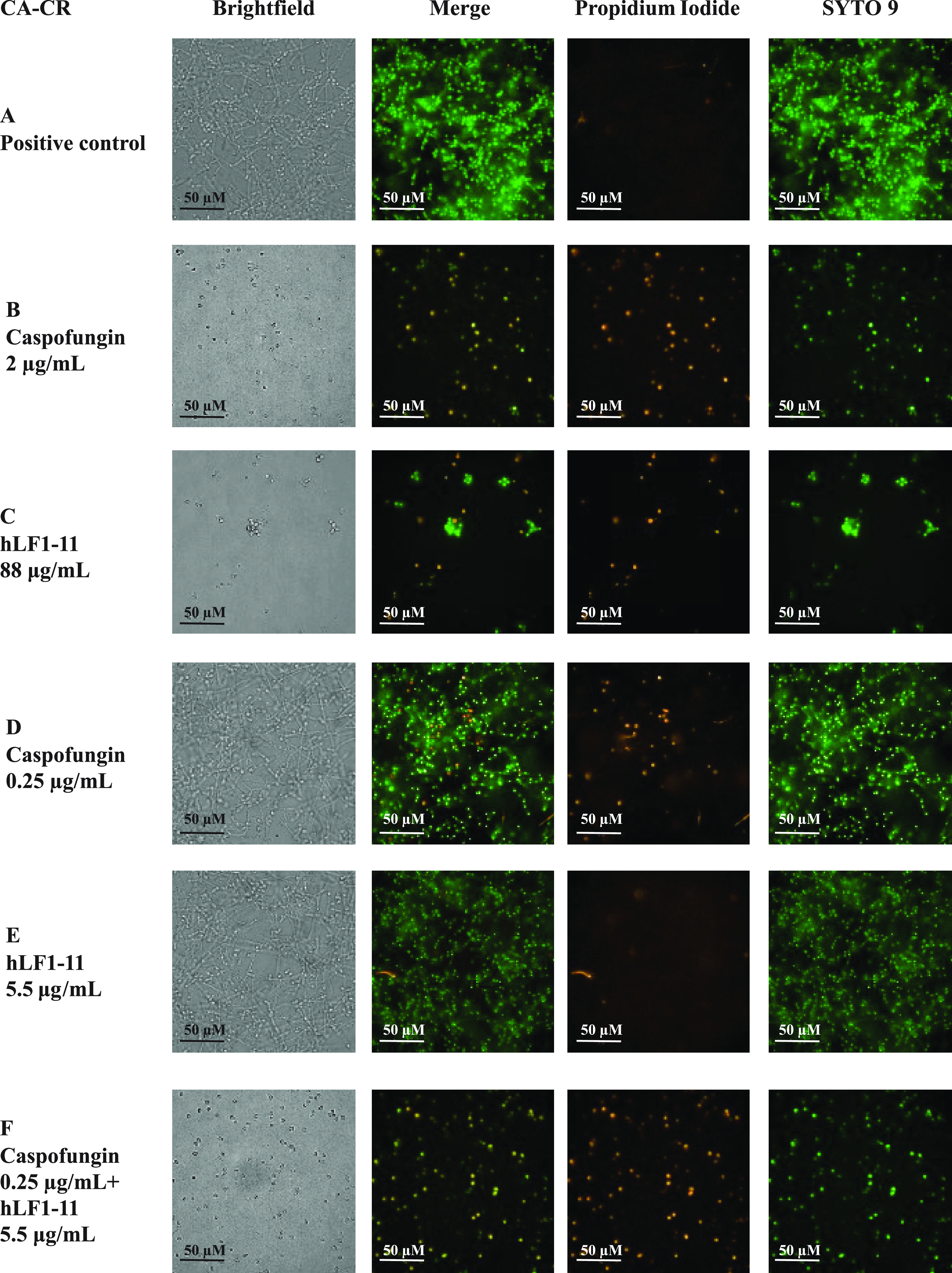
Confocal microscopy images of biofilm produced by C. albicans strain CA-CR. (A) Untreated cells; (B and C) cells treated, in viability assays, with effective concentrations of caspofungin (B) or hLF1-11 (C); (D and E) cells treated with ineffective concentrations of caspofungin (D) or hLF1-11 (E); (F) cells treated with ineffective concentrations of caspofungin and hLF1-11 (F). Size bars, 50 μm.

**FIG 4:**
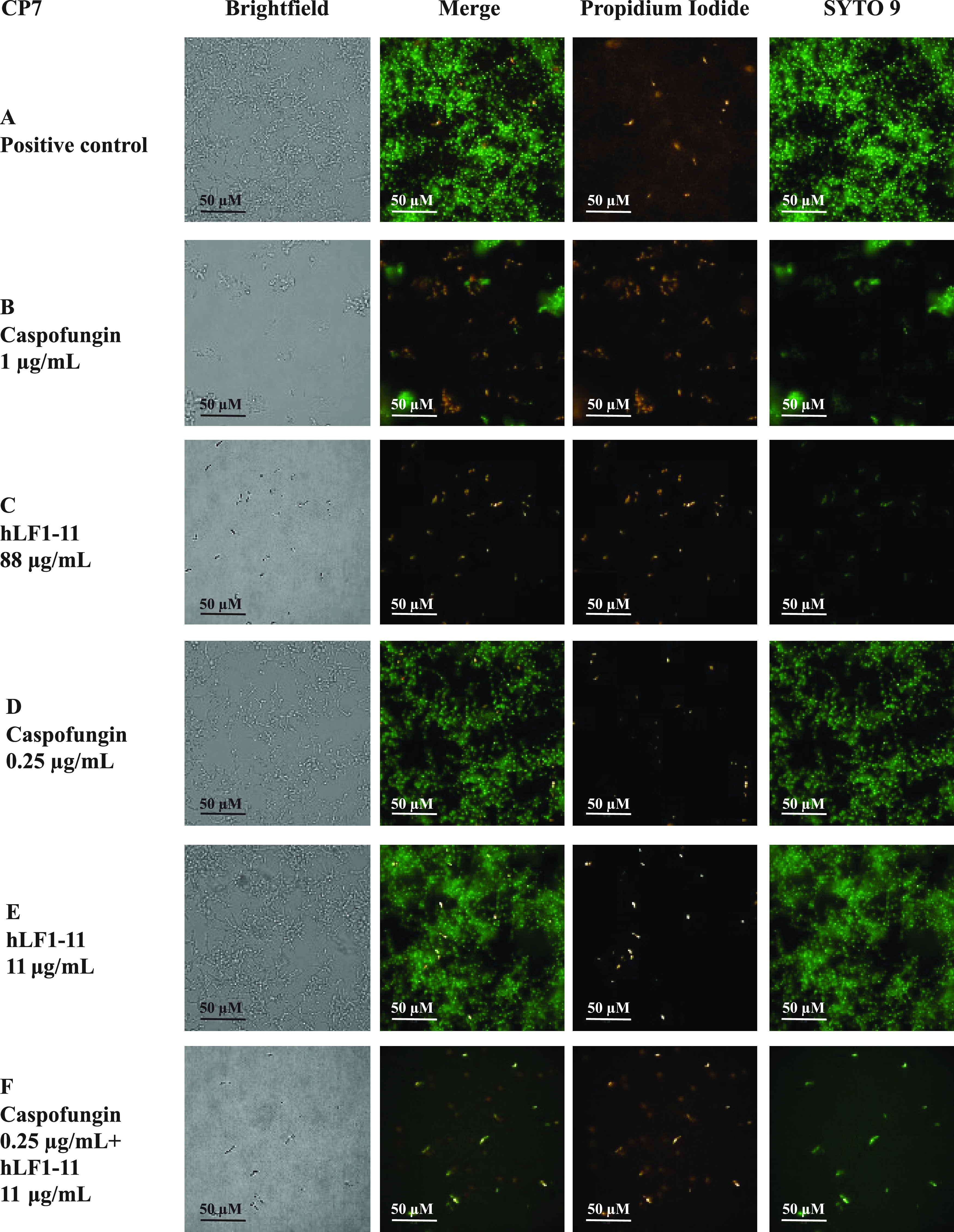
Confocal microscopy images of biofilm produced by C. parapsilosis strain CP7. (A) Untreated cells; (B and C) cells treated, in viability assays, with effective concentrations of caspofungin (B) or hLF1-11 (C); (D and E) cells treated with ineffective concentrations of caspofungin (D) or hLF1-11 (E); (F) cells treated with ineffective concentrations of caspofungin and hLF1-11 (F). Size bars, 50 μm.

**FIG 5:**
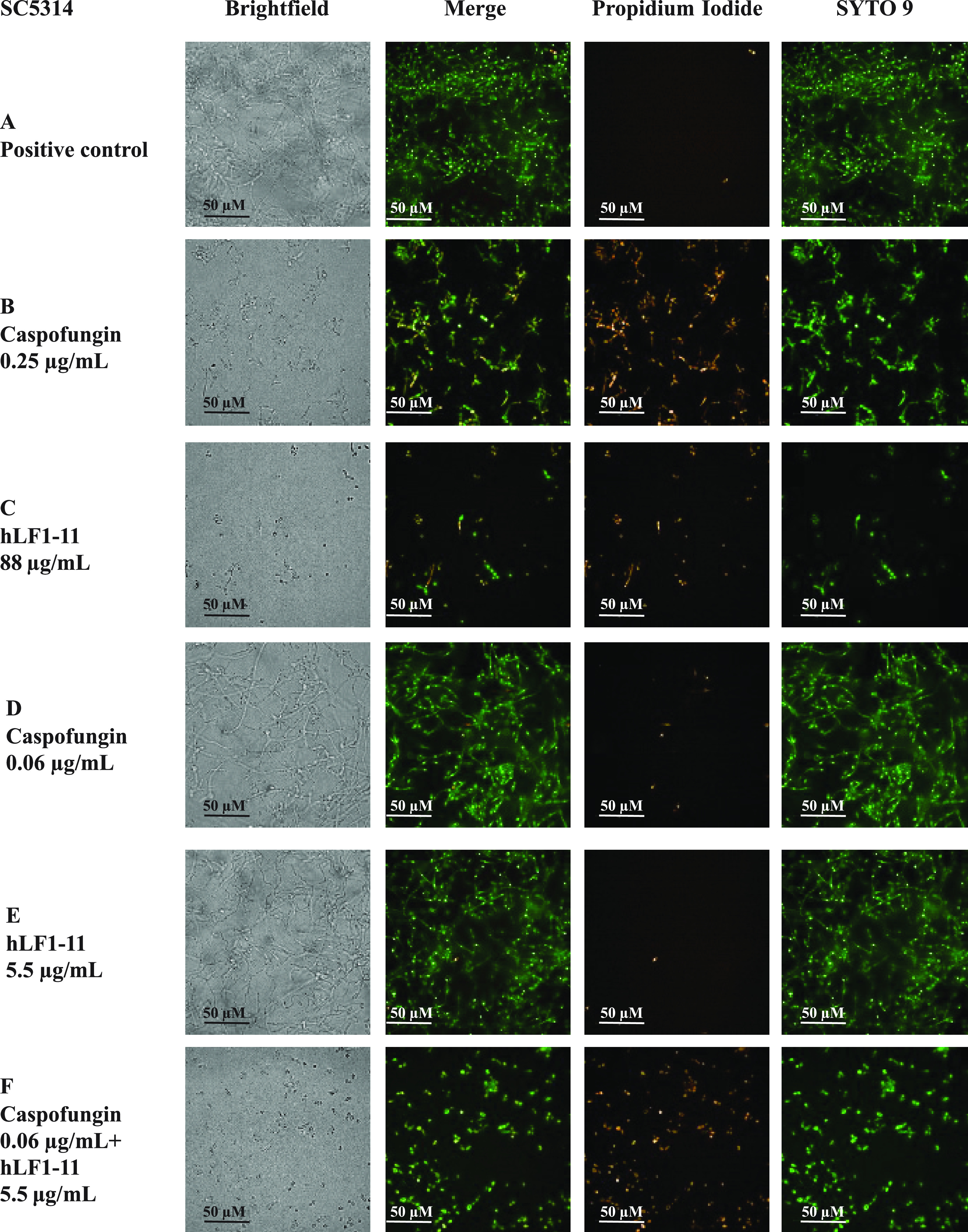
Confocal microscopy images of biofilm produced by C. albicans strain SC5314. A—Untreated cells; (B and C) cells treated, in viability assays, with effective concentrations of caspofungin (B) or hLF1-11 (C); (D) cells treated with ineffective concentrations of caspofungin (D) or hLF1-11 (E); (F) cells treated with ineffective concentrations of caspofungin and hLF1-11 (F). Size bars, 50 μm.

The morphology of biofilm formed by the three previously tested *Candida* strains was observed by confocal fluorescence microscopy. Double staining with SYTO 9 and propidium iodide (PI) allows an estimation of the presence of alive and dead cells, respectively. Strains CA-CR, CP7, and SC5314 produced a thick multilayer biofilm, in which both yeast and hyphal (in C. albicans) or pseudohyphal (in C. parapsilosis) cells could be observed. In all of these strains, both phenotypes of cells are mainly stained with SYTO 9, rarely with PI (Fig. 3A, 4A, and 5A). Cells were treated with the effective concentration of caspofungin and/or hLF1-11, as shown in Fig. 2. Upon treatment, with caspofungin (Fig. 3B, 4B, and 5B) or hLF1-11 (Fig. 3C, 4C, and 5C), cells were mainly in the yeast form and predominantly stained with PI; hyphae or pseudohyphae could not be observed microscopically. Cells treated with ineffective concentrations of caspofungin (Fig. 3D, 4D, and 5D) or hLF1-11 (Fig. 3E, 4E, and 5E) were morphologically similar to the untreated cells (in a thick multilayer, with high cell density, and with a strong presence of hyphal/pseudohyphal cells that mainly stained with SYTO 9, rarely with PI). These ineffective concentrations of hLF1-11 and caspofungin, which exerted a synergistic effect by metabolic and viability assays, were selected. Indeed, the combination of these ineffective concentrations of hLF1-11 and caspofungin allowed us to observe low cell density, with cells mainly stained with PI, and the absence of hyphal and/or pseudohyphal cells (Fig. 3F, 4F, and 5F).

## DISCUSSION

The main conclusion that can be drawn from the present study is that the combination of hLF1-11 and caspofungin exerts synergistic effects under the experimental conditions summarized below. First, a synergistic effect evaluated as metabolic activity reduction and candidacidal activity on planktonic cells restores sensitivity to caspofungin in caspofungin-resistant strains of *Candida* spp. Previous findings demonstrated an antimycotic and antibiofilm effect of hLF1-11 alone on C. albicans or C. parapsilosis (14, 15) and a synergistic effect of hLF1-11 with caspofungin on the planktonic form of C. albicans SC5314 (21).

In the present study, the synergistic effect was obtained on clinical isolates of *Candida* species selected on the basis of caspofungin susceptibility and tested by checkerboard and killing assays. Killing assays performed with ineffective concentrations of hLF1-11 and caspofungin in caspofungin-resistant C. albicans and C. glabrata strains induced more than a 3-log reduction in CFU/mL in comparison to the most active constituent and a 2-log reduction for C. parapsilosis. Much lower drug concentrations were necessary to obtain a synergistic effect on caspofungin-sensitive C. albicans SC5314, in agreement with results previously published by MacCallum et al., obtained under different experimental conditions, such as using malt extract broth (MEB) instead of RPMI diluted 1:4 in sodium phosphate buffer (NaPB) (21). Noteworthy, the combination with hLF1-11 allowed drastic reduction of caspofungin MIC values of caspofungin-resistant isolates, thus reaching values far below the breakpoint for resistance.

Second, the synergistic effect was evaluated as biofilm formation inhibition by a biofilm-producing *Candida* strain.

The combination of hLF1-11 and caspofungin synergistically inhibited biofilm formation. Interestingly, caspofungin BIC values of caspofungin-resistant isolates determined by the checkerboard assay were drastically reduced when caspofungin was combined with hLF1-11. In addition, the cell viability assay confirmed the synergistic effect on the inhibition of biofilm formation for all the tested strains. Further confirmation could be obtained by sample visualization by fluorescence microscopy imaging, performed after a 24-h exposure of cells to hLF1-11 and/or caspofungin. In agreement, the inhibition of the yeast/hyphal transition was previously described in C. albicans after treatment with effective concentrations of hLF1-11 alone in *in vivo* and *in vitro* experiments (14, 19).

To the best of our knowledge, this is the first study exploring the synergistic effect between hLF1-11 and caspofungin on biofilm of various *Candida* species, whereas previous studies have shown synergistic effects by the combination of hLF1-11 with farnesol or nikkomycin on inhibition of C. albicans or C. parapsilosis biofilm formation, with FIC values similar to those reported in the present study (22, 23).

Third, the combination of ineffective concentrations of caspofungin and hLF1-11 was synergistic in decreasing the metabolic activity of mature biofilm, as determined by the checkerboard assay. Moreover, the synergistic concentrations observed by the checkerboard method were further tested in a viability assay. Biofilm was eradicated only for strain SC5314. Although the metabolic activity was significantly reduced upon exposure to ineffective concentrations of hLF1-11 and caspofungin, cells no longer exposed to this drug combination retrieved a replicative phenotype, suggesting a fungistatic rather than a fungicidal effect.

Further studies are needed to better understand the underlying mechanisms involved in the synergistic effect between caspofungin and hLF1-11. Previously, it has been shown that hLF1-11 induced an increase in the mitochondrial membrane’s potential and permeability (17), resulting in the synthesis and secretion of ATP and the production of reactive oxygen species (24), thereby leading to C. albicans cell death. Such a mechanism of action has also been documented by the combination of hLF1-11 and fluconazole (18). Similar results involving reactive oxygen species production and cell membrane permeabilization have been demonstrated for other antimicrobial peptides in yeasts (25, 26).

The present study is based on EUCAST clinical breakpoints that specifically refer to currently recommended therapy dosage (https://www.eucast.org/fileadmin/src/media/PDFs/EUCAST_files/AFST/Clinical_breakpoints/AFST_BP_v10.0_200204_updatd_links_200924.pdf). Synergy could probably lead to dose reduction of each compound. Although previous *in vitro* and *in vivo* studies have shown that hLF1-11 does not exert cytotoxic effects on human erythrocytes and was well tolerated in healthy volunteers with repeated daily doses up to 5 mg (27), further studies are needed to confirm the safety and dosage of the combination of hLF1-11 and caspofungin.

Previous studies showed that hLF1-11 is able to prevent C. parapsilosis and C. albicans (14) biofilm formation and to treat mature C. parapsilosis biofilms grown on PVC (polyvinyl chloride) catheters used for parenteral nutrition. This can be relevant in neonatal intensive care units, where premature newborns are at high risk of developing catheter-related C. parapsilosis systemic infections (28, 29).

In conclusion, the overall findings suggest candidate hLF1-11 as a promising agent to treat recently described caspofungin-resistant *Candida* species isolates.

## MATERIALS AND METHODS

### Strains and growth conditions.

The isolates used in this study are part of a strain collection deposited at the Department of Translational Research, University of Pisa, and stored in YPD broth (yeast extract, peptone, dextrose) (Difco BD, Italy) supplemented with 40% glycerol at −20 and −80°C. Clinical isolates of *Candida* spp. were collected at the Mycology Unit, Azienda Ospedaliero-Universitaria Pisana (Pisa, Italy), identified by matrix-assisted laser desorption ionization time of flight mass spectrometry (MALDI-TOF MS) (Bruker Daltonics, Germany), and tested for antifungal susceptibility according to EUCAST guidelines (http://eucast.org/clinicalbreakpoints/). The strains used in this study were clinical isolates selected on the basis of caspofungin sensitivity and two reference strains (Table 4). For each experiment, cells were inoculated in YPD broth and incubated overnight at 30°C. Next, cells were washed twice in sodium phosphate buffer (NaPB) (0.01 M [pH 7]), and diluted at the desired concentration.

**TABLE 4:** Selected *Candida* strains

Isolate	Origin
C. albicans	
Reference strain SC5314	
Caspofungin-resistant CA-CR	Pharyngeal swab
Caspofungin-resistant CG-C1	Urine
Caspofungin-resistant CG-C2	Central venous catheter
C. parapsilosis	
Reference strain ATCC 22019	
Caspofungin-resistant CP7	Skin

### Antifungal agents.

The synthetic hLF1-11 peptide was purified by Peptisyntha, Inc. (Torrance, CA, USA). hLF1-11 stocks were prepared in NaPB (0.1 M [pH 7]) with 0.01% acetic acid (pH 3.7) at a final concentration of 10 mM and stored at −20°C. Caspofungin (Merck Sharp & Dohme Corp., Inc., USA) was diluted to 1 mg/mL in distilled water and stored at −20°C.

### Checkerboard assay of planktonic cells.

Synergy analyses of hLF1-11 and caspofungin on planktonic cells were carried out by a checkerboard titration method using 96-well round-bottom polystyrene microtiter plates (Sarstedt, Germany). This assay was performed in RPMI 1640 (AppliChem GmbH, Germany) diluted 1:4 in NaPB, with a fungal concentration of 1 × 10^3^ CFU/mL at a final volume of 100 μL, according to the CLSI method (30) for antifungal susceptibility testing. hLF1-11 concentrations ranged from 1.37 to 88 μg/mL, and those of caspofungin ranged from 0.0015 to 2 μg/mL. Each plate included control wells containing the medium alone. After 24 h of incubation at 37°C, the MICs of peptide and caspofungin were defined as the lowest concentration of the agent that produced complete visible growth inhibition. One-dilution variability was considered acceptable for MIC determination. The fractional inhibitory concentration (FIC) index was calculated using the following formula: FIC index = (MIC of drug A in combination)/(MIC of drug A alone) + (MIC of drug B in combination)/(MIC of drug B alone). The FIC indices were interpreted as follows: ≤0.5, synergy; >0.5 and ≤4, no interaction; and >4, antagonism. The FIC index was the mean of the lowest FIC indices from at least three independent experiments.

### Killing assay of *Candida* species planktonic cells.

Killing assays were evaluated in 96-well microplates after exposure to various concentrations of hLF1-11 and/or caspofungin at a final volume of 100 μL. Wells were inoculated with yeast suspensions (10^6^ CFU/mL in RPMI diluted 1:4 in 0.01 M NaPB/well), and wells were incubated at 37°C for 24 h. Viable cells per well were determined by serial dilutions in PBS, plated on YPD agar, and incubated at 30°C for 48 h. Synergy was defined as a ≥2-log-CFU/mL reduction induced by the combination of hLF1-11 and caspofungin, in comparison with its most active constituent (31). All tests were performed in triplicate.

### Checkerboard assay of inhibition of *Candida* species biofilm formation.

Synergy analyses of hLF1-11 and/or caspofungin on 10^6^ CFU/mL sessile cells were carried out by a checkerboard titration method using 96-well flat-bottom polystyrene microtiter plates (Sarstedt, Germany), and biofilm production was evaluated by measuring cellular metabolic activity according to the XTT [2,3-bis-(2-methoxy-4-nitro-5-sulfophenyl)-2H-tetrazolium-5-carboxanilide salt]-menadione assay, performed in RPMI supplemented with 2% glucose and diluted 1:4 in NaPB at a final volume of 100 μL. The hLF1-11 concentrations tested ranged from 1.37 to 88 μg/mL, and those of caspofungin ranged from 0.015 to 2 μg/mL. Each plate included control wells containing the medium alone. After 24 h of incubation at 37°C, wells were washed twice in phosphate-buffered saline (PBS), and the biofilm inhibitory concentration (BIC) was evaluated by XTT assay, as previously described (14). Briefly, XTT solution was prepared at 0.5 g/L in PBS buffer and mixed with a menadione solution dissolved in acetone at a final concentration of 1 mM. An aliquot of XTT-menadione solution (100 μL) was inoculated into each well of a 96-well plate containing dry preformed biofilms and incubated in the dark at 37°C. Following 2 h of incubation, the supernatant (80 μL) was transferred into a 96-well plate to measure colorimetric changes at 490 nm. The BIC was evaluated after background optical density subtraction from each well. The BIC of hLF1-11 and/or caspofungin was defined as at least 50% reduction of metabolic activity compared to the untreated control (14). The FIC index was calculated using the following formula: FIC index = [(BIC of drug A in combination)/(BIC of drug A alone)] + [(BIC of drug B in combination)/(BIC of drug B alone)].

### Viability assay of inhibition of *Candida* species biofilm formation.

The viability of *Candida* spp. in biofilm formation was evaluated in 96-well microplates. Yeast suspensions (10^6^ CFU/mL) in RPMI supplemented with 2% glucose and diluted 1:4 in 0.01 M NaPB/well were incubated with various concentrations of hLF1-11 and/or caspofungin at a final volume of 100 μL. Plates were incubated at 37°C for 24 h and washed twice with PBS to remove nonadherent cells. Sessile cells, detached by scraping with a micropipette tip, were transferred to tubes containing 800 μL PBS, vortexed for 5 min, sonicated (VWR Ultrasonic Cleaner, 230 V/50-60 Hz), vortexed, serially diluted, and plated on YPD agar. CFU were counted after 48 h of incubation at 37°C. Each experiment was performed in triplicate. Synergy was defined as a ≥2-log reduction in CFU per milliliter by the combination treatment in comparison to the most effective compound.

### Synergy studies of *Candida* biofilm morphology.

Visualization of both C. albicans and C. parapsilosis biofilm architecture and inhibition of biofilm formation induced by hLF1-11 and/or caspofungin was performed as previously reported, in glass optically clear, flat-bottom 96-well plates (Perkin Elmer). Following 24 h of incubation at 37°C, wells were washed in PBS and double stained with SYTO 9 (5 nM) and propidium iodide (PI) (30 nM), following the manufacturer’s instructions (Thermo Fisher Scientific, USA).

Biofilms were visualized by confocal fluorescence microscopy (Operetta CLS high-content screening; Perkin Elmer) at a magnification of ×400.

### Data availability.

All data are presented in the article.
